# Proteomic Characterization of Colorectal Cancer Tissue from Patients Identifies Novel Putative Protein Biomarkers

**DOI:** 10.3390/cimb43020074

**Published:** 2021-09-02

**Authors:** Maja Ludvigsen, Louise Thorlacius-Ussing, Henrik Vorum, Mogens Tornby Stender, Ole Thorlacius-Ussing, Bent Honoré

**Affiliations:** 1 Department of Clinical Medicine, Aarhus University, DK-8200 Aarhus, Denmark; majlud@rm.dk; 2 Department of Hematology, Aarhus University Hospital, DK-8200 Aarhus, Denmark; 3 Department of Biomedicine, Aarhus University, DK-8000 Aarhus, Denmark; louise.thorlacius-ussing@regionh.dk; 4 Department of Gastrointestinal Surgery, Aalborg University Hospital, DK-9100 Aalborg, Denmark; mogens.stender@rn.dk (M.T.S.); otu@rn.dk (O.T.-U.); 5 Department of Clinical Medicine, Aalborg University, DK-9100 Aalborg, Denmark; henrik.vorum@rn.dk; 6 Department of Ophthalmology, Aalborg University Hospital, DK-9100 Aalborg, Denmark

**Keywords:** colorectal adenocarcinoma, colorectal cancer, protein expression, proteomics, biomarkers

## Abstract

Colorectal cancer (CRC) is one of the leading causes of cancer-related death over the world. There is a great need for biomarkers capable of early detection and as targets for treatment. Differential protein expression was investigated with two-dimensional gel electrophoresis (2D-PAGE) followed by identification with liquid chromatography–tandem mass spectrometry (LC-MS/MS) in CRC patient tissue from (i) the peripheral part of the tumor, (ii) the central part of the tumor as well as from (iii) a non-involved part of the colorectal tissue. The expression patterns of six identified proteins were further evaluated by one-dimensional Western blot (1D-WB) analysis of the CRC tissue. Proteins that were perturbed in expression level in the peripheral or in the central part of the tumor as compared with the non-involved part included *S100A11*, *HNRNPF*, *HNRNPH1* or *HNRNPH2*, *GSTP1, PKM* and *FABP1*. These identified markers may have future diagnostic potential or may be novel treatment targets after further evaluation in larger patient cohorts.

## 1. Introduction

Colorectal cancer (CRC) is one of the leading causes of cancer-related death over the world. Therefore, considerable resources are spent on identifying protein biomarkers for diagnostic, prognostic and therapeutic purposes. Various models based on “omics” techniques have been used in the past couple of decades [1], and proteomics techniques, especially for the past decade [2], were used for the purpose of identifying markers of CRC based on various sources such as cell cultures, organoids as well as primary tumor tissue [2]. Recently, we evaluated a biomarker discovery approach consisting of proteomic analysis of a two-dimensional cell culturing model system followed by analysis of patient colorectal cancer tissue [3]. The system consisted of a normal derived colon mucosa cell line, NCM460 [4], analyzed against two different colorectal cancer cell lines established from human colonic carcinoma [5,6]. The obvious advantage of such a system is the access to virtually unlimited amounts of homogeneous samples for the initial discovery based proteomic analysis identifying differentially expressed proteins, i.e., putative biomarkers that subsequently can be further analyzed in patient tissue. However, in spite of the obvious advantages of a homogeneous cellular model system, one of the disadvantages of such a model is that there exist a number of different cell lines with different growth potentials [7]. Furthermore, cultured cell lines may change phenotype during culturing [8]. This may result in a long list of putative markers that need to be tested afterwards on patient samples and proteins may also be missed in the initial screening. The cell culturing can be further developed to three-dimensional cell culturing where spheroids and organoids can be used to approach the cell culture system to mimic the structural features of the tumor [2]. However, the tumor tissue remains the primary source to accurately represent the CRC proteome [2].

Analysis of colorectal tumor tissue from patients is inherently complicated due to both *intra* tumor [9,10] as well as *inter* tumor heterogeneity [10]. Moreover, even normal colon tissue exhibits some degree of protein expression heterogeneity along the intestinal tract [11]. Detailed protein analysis such as protein phosphorylation is even more complicated due to slight variations in sampling conditions [12]. Thus, resected colorectal tumors from patients may possess heterogeneity due to *inter* and also *intra* biological differences within the tumor from the central parts that may show some degree of necrosis to the peripheral parts that also may contain normal cells by being close to the non-involved colorectal tissue. Despite of all these concerns with tissue heterogeneity, we decided to try whether our previously described 2D-PAGE technology [3] in a simplistic approach applied directly to different parts of the tumor with subsequent immunological verification (1D-WB) on the same tissue could bring novel putative markers to attention. From ten patients, we analyzed samples collected from both the central part of the tumor as well as from the peripheral part of the tumor and compared each of them with the non-involved colorectal tissue from the furthest resection margin to identify differences in protein expression. Furthermore, differential protein expression was correlated with the TNM stage.

Although heterogeneity certainly is a concern, several perturbed protein spots that were observed centrally as well as peripherally with the 2D-PAGE technique possessed no major discrepancies in the observed differential expression levels. Only few and slight differences were observed between tumors when TNM high stage tumors were compared with low stage tumors. Some of the identified proteins observed here have already been investigated as promising while others need further investigation for suitability as biomarkers or drug targets.

## 2. Materials and Methods

### 2.1. Tissue Biopsies from Patients with CRC

Tissue samples were obtained from ten patients with CRC. Previously, we have performed analyses with samples from this patient cohort and protein extraction was carried out as described [3]. In short, 3 samples were collected from each patient, one from the central part, one from the peripheral part of the tumor and one from the resection margin furthest from the tumor. The resected tissue was rinsed in cold isotonic sodium chloride and the excised biopsies were frosen in liquid nitrogen before being stored at −80 °C. Upon thawing the tissue was rinsed with PBS-buffer and then solubilized in IP 3-10 NL lysis buffer (9M Urea, 2% (*v*/*v*) Triton X-100, 2% (*w*/*v* DTT, 2% (*v*/*v*) IPG-buffer (GE Healthcare, Chicago, IL, USA) using a homogenizer. Protein concentration was determined using the Non-Interfering Protein Assay (488250, Calbiochem^®^, Merck KGaA, Darmstadt, Germany).

### 2.2. Two-dimensional Polyacrylamide Gel Electrophoresis (2D-PAGE) and Liquid Chromatography–Tandem Mass Spectrometry (LC-MS/MS) for Protein Identification

The 2D-PAGE was essentially performed and stained as previously described [3] with the modification that gels were scanned using an ImageQuant LAS4010 (GE Healthcare, Cleveland, OH, USA) and the generated TIFF files were imported into PDQuest version 8.0 [13]. Quantification of the single protein spot was performed relatively by normalizing the volume of each protein spot detected in the gel to the total volume of all the protein spots detected in the gel. Significantly differentially expressed spots were detected with a fold-change >1.5. The significant changes (*p* < 0.05) were detected using a Wilcoxon signed rank test to detect differences between peripheral (P), central (C) and non-involved part of the colon (N) and a Mann–Whitney U-test was used to detect differences between different TNM stages of the tumors. Differentially expressed spots were manually cut out and subjected to *in-gel* tryptic digestion and LC-MS/MS, as described [3]. Proteins were identified by searching in the SwissProt protein database (releases 2011_08, 2012_02 and 2012_04) using the online version of Mascot MS/MS ions Search facility (Matrix Science, Ltd., London, UK) [14]. Doubly and triply charged ions with up to two missed cleavages were included in the analysis. The peptide tolerance was set to ±20 ppm and the fragment mass tolerance to ±0.05 Da. A variable modification was included, carbamidomethyl (C) and occasionally oxidation (M). Contaminating peptides, including keratins, trypsin and casein, were disregarded. Ion scores were derived as −10Log(*p*) and values for “bold red” peptides (peptides with the most logical assignment to the proteins) above approximately 35–37 indicated identity or extensive homology with less than 5% probability that the match was a random event.

### 2.3. Western Blotting

Western blotting and quantitation of bands were performed as previously described [3]. To each lane, an equal amount of protein was loaded and the total protein level was used for normalization of the blots rather than using household proteins for the reasons we [3,15] and others [16,17] have previously given, that the ideal reference gene or protein is still missing. A Wilcoxon signed rank test was used to identify significant differences as previously described [3].

Primary antibodies, anti-hnRNP F and anti-hnRNP H1/2, were affinity-purified polyclonal antibodies generated and characterized in our laboratory [18]. The other antibodies were purchased from commercial suppliers: rabbit anti-S100A11 (ab97329, Abcam, Cambridge, UK), mouse anti-FABP1 (ab82157, clone 2G4, Abcam), rabbit anti-PKM (HPA029501, Atlas Antibodies, Merck, Darmstadt, Germany) and rabbit anti-GSTP1 (HPA019779, Atlas Antibodies). Secondary HRP-conjugated antibodies were rabbit anti-mouse PO260 HRP and swine anti-rabbit PO217 HRP, purchased from Dako Denmark A/S (Glostrup, Denmark).

## 3. Results

### 3.1. Patient Characteristics

Tissue samples were excised from patients with CRC during intended radical surgery. From each patient, three samples were collected: (i) one from the peripheral part of the tumor, (ii) one from the central part of the tumor as well as (iii) one from the resection margin furthest from the tumor. 

The patient characteristics have previously been given [3] and are listed in Table 1.

### 3.2. Protein Expression Patterns in the Central versus the Peripheral Part of the Tumor

**Figure 1:**
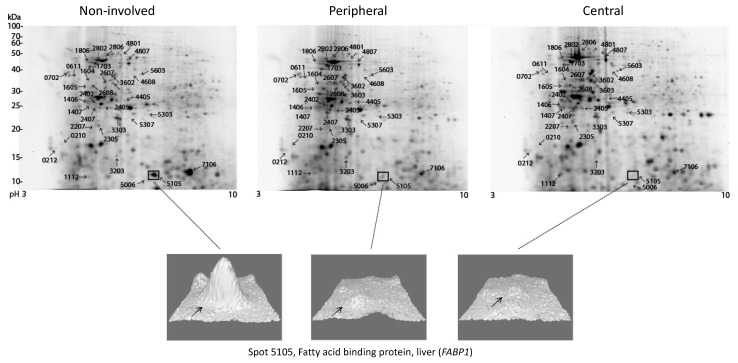
Proteomic analysis of patient colorectal tissue obtained from non-involved (N), peripheral (P) and central (C) part of the tumor. Comparative analysis of the spots was performed with the PDQuest software. Differentially expressed spots with a change of 1.5-fold or more are shown (Wilcoxon signed rank test, *p* < 0.05). Identification of differentially expressed proteins are listed in Table 2.

The expression patterns in the central (C) and the peripheral (P) part of the tumor together with the non-involved (N) part of the colorectal tissue were investigated by 2D-PAGE in order to find differences in protein expression, i.e., at least 1.5-fold significant differences (Wilcoxon signed rank test, *p* < 0.05), Figure 1. 

Differentially expressed spots between the compared groups are indicated in Figure 1. Specific proteins were identified from the excised spots by LC-MS/MS, as listed in Supplementary Table S1. The various comparisons in which the spots were identified as differentially expressed are listed in Table 2 together with the identifications. For simplicity, the protein identifications are mostly listed and described using the gene names, since in most cases, it was not possible to distinguish between different proteoforms [19] that may occur as a result of, e.g., alternative splicing. Several of the proteins were identified from spots with lower molecular masses than the theoretical masses, indicating that the identified spot contains a fragment (*fr*) of the protein. In such cases, it cannot be firmly concluded from an upregulated spot containing a protein fragment whether the protein is up- or downregulated but merely concluded that the protein level is perturbed. In some cases, there was more than one identification in one spot, and then it was impossible to establish which of the proteins were accountable for the observed difference in protein expression. 

### 3.3. Proteomic Comparison of Various Parts of the Tumor with Non-involved Colorectal Tissue 

High expression in the central part of the tumor compared with the non-involved part was found in 17 spots, e.g., *TUBB2A fr* (spot 0212), *TXN* (spot 1112), *ALB fr* (spot 1406), spot 1407, *ACTB fr* (spot 1605), spot 1806, *HNRNPF fr* (spot 2405), spot 2407, spot 2607, *betaTub56D fr* (spot 2608), spot 3303, spot 3602, spot 4405, *S100A11* (spot 5006), spot 5303, spot 5307 and *PKM fr* (spot 5603). Of these 17 spots, nine were also found to be upregulated in the peripheral part of the tumor while one additional spot (4608) was detected as upregulated in the peripheral part of the tumor.

Four spots were found with low expression in the central part of the tumor compared to the non-involved part, including *MYL6* (spot 0210), spot 2305, spot 4807 and *FABP1* (spot 5105). Only the latter of these, *FABP1,* was also detected in the peripheral part of the tumor, while one additional spot was detected as downregulated in the peripheral part of the tumor, *LUM* (spot 4801); see Table 2 and Figure 1.

The 2D-PAGE analysis identified most differentially expressed proteins in relation to non-involved colorectal tissue when the central part of the tumor was analyzed as compared with the peripheral part of the tumor. Few additional differentially expressed proteins were observed in the peripheral part of the tumor. Thus, there may be a certain degree of heterogeneity in the tumor. However, only few spots, 0611 and 1605, showed differential expression between the peripheral part versus the central part of the tumor and moreover, when the proteins were detected as differentially expressed in the central part as well as in the peripheral part, the fold-changes observed were overall very similar (Table 2).

### 3.4. Protein Expression Patterns in High TNM Stage versus Low TNM Stage

Patients were grouped according to TNM classification (Table 1) in a low stage group, TNM_L_ (five patients), without involvement of regional lymph nodes and without distant metastases and a high stage group, TNM_H_ (five patients), with involvement of either regional lymph nodes or distant metastases as given in Table 1. Six spots were identified as differentially expressed when analyzing the samples from the peripheral part of the tumor according to tumor stage. All spots in the tumor showed lowest expression in TNM_H_, e.g., *ATP5F1B fr* (spot 1604), *HSP8A* (spot 1703), spot 2207, *actb* (spot 2802), spot 2806 and spot 3603, Table 2. None of these were seen in the central part of the tumor where two other spots were detected, spot 3203 and *HBB* (spot 7106). In the non-involved colorectal tissue two spots were found to be differentially expressed, e.g., *SERPINA1* (spot 0702) and spot 2402, Table 2. 

Thus, the 2D-PAGE analysis revealed only minor differences in expression levels with stage, mostly observed in the peripheral part of the tumor and generally with downregulation in the high stage group. None of the spots were shared between the analyses.

### 3.5. Immunological Evaluation of Identified Differentially Expressed Proteins

**Figure 2:**
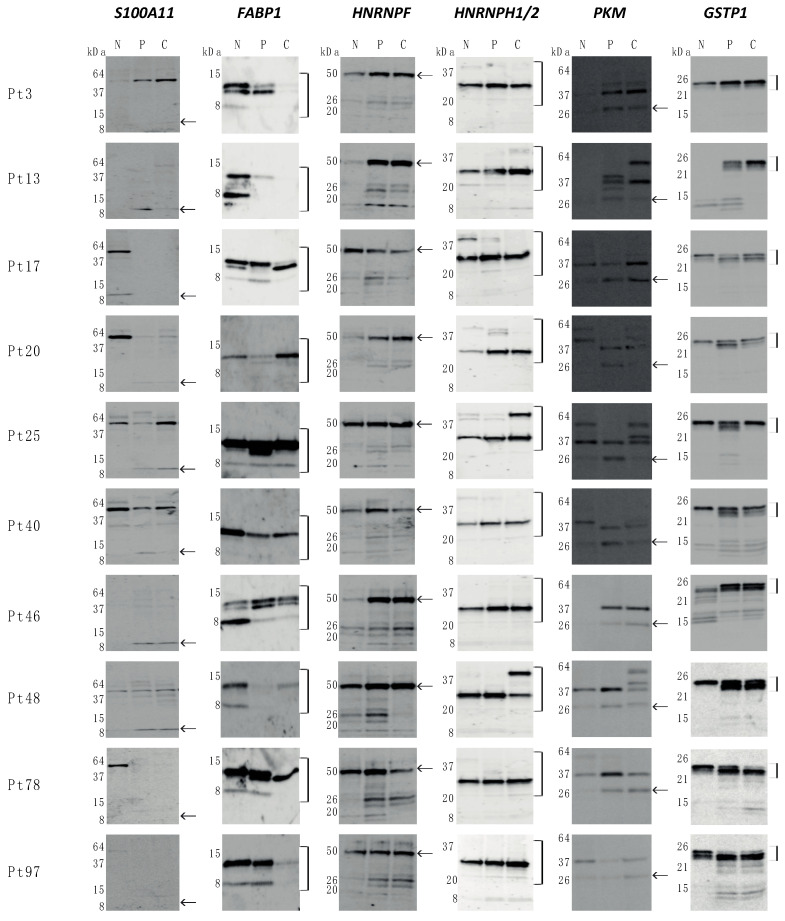
Western blotting of tissues from CRC patients from non-involved part of the colorectal tissue (N), peripheral part of tumor (P) and central part of tumor (C). Antibodies against *S100A11*, *FABP1*, *HNRNPF*, *HNRNPH1/2*, *PKM*, and *GSTP1* were incubated with the blots. Total pixel intensity was measured in the bands as indicated to the right of the blots. The value of the bands in N was set to 1 and the P and C samples were calculated relative to the N value and log_2_-transformed as given in Figure 3.

**Figure 3:**
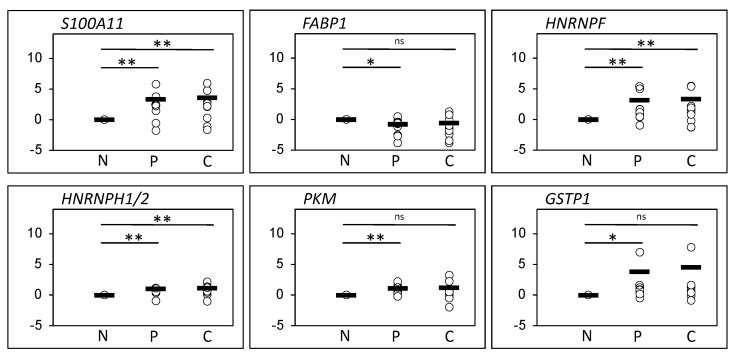
Quantification of *S100A11*, *FABP1*, *HNRNPF*, *HNRNPH1/2*, *PKM* and *GSTP1*. Relative pixel intensities are depicted as log_2_-transformed values in the dot plots. Quantification was performed as indicated in the legend to Figure 2. * *p* < 0.05; ** *p* < 0.01, ns; not significant.

The expression pattern of a number of the differentially expressed proteins identified from the 2D-PAGE were further evaluated by 1D Western blotting (1D-WB) of the peripheral and the central part of the tumor compared with non-involved colorectal tissue from the CRC patients (Figure 2 and Figure 3).

Significant differential expression was observed by 2D-PAGE for *S100A11* identified from spot 5006 in the central part of the tumor compared with the non-involved part (Figure 1 and Table 2). *S100A11* has an expected molecular mass of 11.7 kDa and assessing the protein expression in the band corresponding to this molecular mass in 1D-WB analysis confirmed the differential expression together with differential expression between the central as well as the peripheral tumor tissue versus the non-involved colorectal tissue (both *p* < 0.01) (Figure 2 and Figure 3). The antibody recognizes various additional bands with higher molecular masses, indicating that *S100A11* may show dimerization/multimerization or non-specific reaction.

Low expression was observed for *FABP1* (spot 5105) in both the central and the peripheral part of the tumor compared with the non-involved part (Figure 1). In 1D-WB analysis, two closely migrating bands corresponding to the expected molecular mass of *FABP1* (14.2 kDa) were found together with a band of an even lower molecular mass. A significant difference in expression was identified between the peripheral part of the tumor (P) compared with the non-involved part (N) on basis of expression from either all the bands in total or the two co-migrating bands (both *p* < 0.05) (Figure 2 and Figure 3).

From the 2D-PAGE proteomic comparison, spot 2405, *HNRNPF fr*, was found with high expression in both the central part and the peripheral part of the tumor compared with the non-involved part (Figure 1 and Table 2). From this spot, a fragment of *HNRNPF* (*HNRNPF fr*) was identified on the basis of two peptides. However, this spot migrates with a lower molecular mass (approximately 30 kDa) than the expected molecular mass of *HNRNPF* (45.7 kDa). Moreover, 1D-WB analysis enabled the quantification of full-length *HNRNPF,* confirming the differential expression with high expression in the central as well as the peripheral part of the tumor tissue as compared with the non-involved colorectal tissue (*p* < 0.01, *p* < 0.01) (Figure 2 and Figure 3). Various bands with lower molecular masses were also recognized by the *HNRNPF* antibody.

Fragments of *HNRNPH1/2 (HNRNPH1/2 fr*) were identified from two spots (5303 and 5307), both migrating with a lower molecular mass than expected for the un-cleaved *HNRNPH1/2* and both spots contained additional peptides identifying other proteins, i.e., *RAB11A*/*B* (spot 5303) and a collagen chain fragment *COL1A2* (spot 5307) (Figure 1 and Table 2). Spot 5303 was identified with high expression in the central tumor tissue compared with non-involved colorectal tissue, whereas spot 5307 was identified with high expression in both tumor tissue samples (Table 2). With 1D-WB analysis, expression of *HNRNPH1/2* was observed in all samples though in bands migrating with lower molecular masses (approximately 30 kDa) than the expected (49 kDa) for the un-cleaved protein. Quantification of all bands confirmed the high expression in the tumor tissue, both central and peripheral compared with the non-involved colorectal tissue (*p* < 0.01, *p* < 0.01) (Figure 2 and Figure 3). 

Fragments of *PKM* (*PKM fr*) were identified from spot 5603, from spot 4405 together with *PRDX4* and *ATP5F1B fr* and from spot 4608 together with *TCP1.* Spot 4405 showed higher expression in the peripheral and central part of the tumor compared to the non-involved part, whereas spot 5603 only was identified as differentially expressed in the central tumor tissue compared with the non-involved colorectal tissue. Spot 4608 was differentially expressed in the peripheral part of the tumor. All three spots migrated with lower molecular masses (approximately 25–40 kDa) than the expected mass of *PKM* (58 kDa). With 1D-WB analysis, various bands were observed. Significant differential expression was identified from quantifying the band of approximately 25 kDa, which showed a high expression in the peripheral part of the tumor in comparison with the non-involved part (*p* < 0.01) (Figure 2 and Figure 3). 

From spot 3303, *GSTP1* and *PRDX2* were identified together with fragments of *CRKL* and *EZR*. This spot showed high expression in the tumor tissue, both in the peripheral and in the central part compared to the tissue from the non-involved part. Both *GSTP1* and *PRDX2* were evaluated with Western blot analysis, showing no significant correlation for *PRDX2*. In contrast, *GSTP1* expression showed high expression in the peripheral part of the tumor compared with the non-involved part. Quantification was carried out on the basis of the two closely migrating bands corresponding to the molecular mass, 23 kDa, of *GSTP1* (*p* < 0.05) (Figure 2 and Figure 3).

With the 1D-WB analysis, all of the six proteins or fragments analyzed, *S100A11*, *FABP1*, *HNRNPF*, *HNRNPH1/2*, *PKM* and *GSTP1*, showed perturbation of the expression in the peripheral part of the tumor while a similar difference in the central part of the tumor was only detected with three of the proteins. No significant differential expression was observed with 1D-WB of *TXN* (spot 1112), *ATP5F1B* (spots 1604, and 4405), *SERPINA1* (spot 0611 and 0702), *HSP8A* (spot 1703 and 1806) and *PRDX2* (spot 3303). 

Since heterogeneity of samples may be a problem, we also analyzed the 1D-WB by omitting the rectal sample and keeping the nine colon samples. Although slight changes were observed, we found largely similar significant changes as with the 10 included patients (Figure 3), except that the C versus N analysis was also significant with *FABP1* and the P versus N analysis did not retain its significance with *HNRNPF*.

## 4. Discussion

All tissue samples for the present study were collected at time of CRC surgery and sampled from the central and the peripheral part of the tumor together with tissue sampled from a non-involved part of the colorectal tissue furthest away from the tumor. Using 2D-PAGE, we identified several differentially expressed protein spots. The 2D-PAGE technology possesses a number of advantages as well as disadvantages. An advantage is that it is able to separate various proteoforms in spite of the fact that the chemical differences between the proteoforms are unknown. This should be kept in mind when differentially expressed protein spots detected by 2D-PAGE are immunologically verified using 1D-WB. A protein, such as triosephosphate isomerase, does not change level by 1D-WB of normal derived colon cells compared with colon cancer cells [3]. However, the protein separates in at least three proteoforms, upregulated as well as downregulated by 2D-PAGE. This can explain apparent discrepancies observed in fold changes between 2D-PAGE and 1D-WB. When fragments of proteins are identified as differentially expressed by 2D-PAGE, it is not possible to firmly establish whether the protein is up- or downregulated, but merely that the level is perturbed. Another issue is that a spot observed by 2D-PAGE may occasionally contain more than one identified protein and in such a case, it may not be obvious which of the proteins are responsible for the observed fold change. If one of the proteins are identified with several more peptides than others, it may be a good candidate. Analysis of colorectal cancer is further complicated by the heterogeneity of normal tissue along the intestinal tract [11] together with *inter* tumor heterogeneity [10] as well as *intra* tumor heterogeneity [9,10]. In spite of this, we observed several differentially regulated spots in 2D-PAGE when tumor tissues especially from the central part and also to a certain degree from the peripheral part were compared with non-involved colon tissue. Some of these proteins were also found to be differentially expressed with 1D-WB. With the latter technique, most of the differentially expressed proteins were detected in the peripheral part of the tumor. A number of the detected proteins here have previously been observed as strong candidates for CRC biomarkers, supporting the validity of the detection system.

Several studies have shown that *S100A11* is upregulated in colorectal cancer tissue [20,21,22]. Recently, Zeng et al. used bioinformatic tools to analyze several S100 transcripts and found that the *S100A11* transcript was significantly upregulated in CRC tissue compared with normal colon mucosa [23]. Moreover, they found that *S100A11* together with *S100A1* and *S100A2* was significantly correlated with CRC stage and progression, suggesting that they are among potential prognostic markers [23]. Recently, Guo et al. also found that the protein *S100A11,* amongst others, was upregulated in both non-metastatic and metastatic CRC tissue [24]. Our proteomic results are fully in line with these observations and upregulation was detected with 2D-PAGE in the central part and with 1D-WB analysis in the central as well as in the peripheral part. Although *S100A11* is upregulated in CRC tumor tissue, the serum level of *S100A11* is lower in CRC patients [25].

*FABP1* was previously reported to be consistently downregulated in 20 colorectal cancer samples by 2D-PAGE analysis [26]. Recently, Zhang et al. analyzed the transcript levels of *FABP1* in CRC by single-cell sequencing and found that the *FABP1* transcript was the 10th transcript amongst the 20 most discriminative transcripts in 272 colorectal cancer epithelial cells compared with 160 normal epithelial cells [27]. Our 2D-PAGE analysis showing downregulation of the *FABP1* protein in the central part as well as in the peripheral part is perfectly in line with this. With 1D-WB analysis, we only detected significant downregulation in the peripheral part of the tumor.

We previously analyzed the expression levels of *HNRNPF* and *HNRNPH1* or *HNRNPH2* on normal and several types of cancer tissues [28]. The expression levels of the proteins in normal colonic epithelium of the gastrointestinal tract ranged from not detectable up to moderate expression, while moderate to very strong cytoplasmic expression was seen in rectal carcinoma [28]. In the present study, we observed a number of fragments with 2D-PAGE as well as with 1D-WB analysis. This is fully in line with our previous observations on various tissues [28]. Recently, Takahashi et al. showed that *HNRNPH1* is highly upregulated in colorectal cancer cell lines, inhibits apoptosis and promotes colorectal cancer progression through stabilization of the mRNA for sphingosine-1-phosphate lyase 1 [29]. Xu et al. have shown that all three proteins associate with the human telomerase RNA component and with the telomerase holoenzyme to regulate telomerase activity and play important roles in modulating telomerase activity and telomere length. Moreover, deletion of the proteins impairs cancer and stem cell proliferation and induces senescence, while overexpression delays stem cell senescence [30]. Our 2D-PAGE revealed some fragments to be upregulated while 1D-WB showed upregulation of *HNRNPF* and perturbation of *HNRNPH1/2* in the central as well as in the peripheral part of the tumor. Taken together, these observations make *HNRNPH1, HNRNPH2* and *HNRNPF* strong candidates as biomarkers or drug targets for CRC.

There are four isozymes of pyruvate kinase, L, R, M1 and M2 [31]. The four isozymes are encoded by two different genes. *PKLR* encodes L and R by using different promoters [32] while the *PKM* gene encodes the two alternatively spliced isozymes, M1 and M2 [33]. Kim et al. have, by immunohistochemistry, shown that the M2 form is upregulated in CRC as compared with normal colon tissue [34]. Moreover, substantial genomic analysis of reported gene expression data revealed that the *PKM* transcript expression was significantly higher in colon tissue from CRC patients than in normal tissue in several patient cohorts and that patients with high *PKM* transcript levels had poor clinical outcome [34]. Our 2D-PAGE analysis showed differential expression of spots containing *PKM fr* in the central part and 1D-WB analysis showed perturbation of the expression in the peripheral part of the tumor. Indeed, this brings *PKM* among the candidates as biomarkers for CRC. As a drug target, however, things are much more complicated as depletion of the M2 isoform increases the level of M1 and it seems that deficiency of M2 in intestinal stem cells accelerates progression of colorectal cancer in a murine model [34].

Glutathione S-transferases consists of three major classes of isozymes, α, μ and π (*GSTP1*). *GSTP1* is the predominant form in colon tissue [35]. Several studies have found *GSTP1* to be upregulated in colon cancer in comparison with normal colon tissue using immunohistochemistry [35,36,37,38] and Western blotting [39]. We found spot 3303 with *GSTP1* was upregulated in the central as well as the peripheral part of the tumor and by 1D-WB, *GSTP1* was upregulated in the peripheral part. Thus, *GSTP1* is a strong biomarker candidate for CRC.

In our previous study on CRC tissue [3], we also detected a number of other proteins such as members of the CREC proteins, *S100A6* and *SET* (Protein SET) as putative markers using the same patient cohort. Of the CREC proteins, *CALU* is under investigation where, together with *AURKA* and *MCM2*, it could successfully distinguish tumors from healthy samples [40]. We detected *CALU* to be upregulated in the central as well as in the peripheral part of the tumors [3]. *RCL1* has not been thoroughly studied with respect to CRC. We previously found it to be upregulated in the peripheral as well as in the central part of the tumors by 1D-WB [3]. In our cellular model systems, *RCL2* (ERC-55) did not show significant changes in the tumor cells [3]. However, *RCL2* has recently been demonstrated to be upregulated in colorectal cancer and associated with disease-free survival and overall survival [41]. High *RCN2* expression was an independent prognostic marker of poor outcome [41]. *S100A6* has previously been shown to be upregulated in colorectal adenocarcinomas [42]. To the best of our knowledge, *SET* has not been thoroughly investigated with respect to CRC.

It is important to note that our study possesses some limitations. First, 2D-PAGE is a labor-intensive technique both with respect to the laboratory handling as well as the software analysis, so to keep the analysis as simple as possible, we limited the study to ten patients which, however, is a small sample size. In addition, one rectal sample was included among nine colon samples. This might be a challenge due to the heterogenous nature of the gastrointestinal tract and the radiotherapy that might affect the protein expression. It was reassuring, however, that elimination of this single sample from the ten samples showed an overall similar tendency, having in mind that the statistics also may change simply because of a reduction in the sample size. Another limitation is that the immunologic verification was performed on the same biologic material. Finally, a number of statistical tests were performed, and correction for multiple hypothesis testing could be warranted in order to reduce type 1 errors. However, this would be at the expense of increased type 2 errors with the risk that putative novel markers unfortunately may be missed. Since the present analysis is a discovery-based, hypothesis-generating study bringing attention to novel putative markers, we abstained from such a correction. We emphasize that the suggested set of biomarkers needs to be verified on a different and larger cohort of patients.

## 5. Conclusions

The simplistic approach presented here, although not ideal, seems to be able to detect at least some biomarkers in the peripheral part or in the central part of the tumor, depending on the method of detection. Only minor differences were detected according to stage of tumor. The applicability of these markers should be further investigated in order to establish their potential role as CRC biomarkers or drug targets.

## Figures and Tables

**Table 1:** Patient characteristics.

Patient ID No.	Sex	Age by the Time of Diagnosis	Localization	T	N	M	TNM-Stage	Rt	Recurrence	Death Due to CRC
3	M	63	Colon	4	2	1	High	No	NA	Yes
13	M	73	Colon	3	1	1	High	No	NA	Yes
17	M	58	Colon	4	2	0	High	No	Yes	Yes
20	F	75	Rectum	2	0	0	Low	Yes	No	-
25	M	65	Colon	3	0	0	Low	No	Yes	-
40	M	69	Colon	2	0	0	Low	No	No	-
46	M	76	Colon	3	0	0	Low	No	Yes	-
48	F	80	Colon	3	0	1	High	No	NA	Yes
78	F	54	Colon	4	1	1	High	No	NA	Yes
97	M	79	Colon	3	0	0	Low	No	No	-

Characterization of patients included in the project. Sex: Female (F)/Male (M); Age by the time of diagnosis: in years; TNM Clinical Classification: T = primary tumor 1–4, N = Regional Lymph Nodes 0–2, M = Distant Metastasis 0–2; Rt, Radiotherapy: Yes = Short-course 25Gy/5 fractions; Recurrence: NA = not appropriate (due to initial M1 status). Data are previously published [3].

**Table 2:** Identification of differentially expressed proteins in tumor tissue from CRC patients.

		Fold Change					
Spot No.	Protein ID (Gene)	C/N	P/N	P/C	TNM_H_/TNM_L_		
					P	C	N
0210	*MYL6*	0.44					
0212	TUBB2A fr *	3.20	3.50				
0611	*SERPINA1 fr* *HSP90B1 fr or* *HSP90AB1 fr*			2.43			
0702	*SERPINA1 fr*						0.53
1112	*TXN*	3.19	2.84				
1406	*ALB fr*	2.07					
1407	*ACTB fr;* *ENO1 fr;* *ALB fr;* *FGG fr*	3.72	3.37				
1604	*ATP5F1B fr*				0.50		
1605	*ACTB fr*	3.73		5.27			
1703	*HSP8A fr*				0.35		
1806	*HSP8A fr;* *VIM fr* *HSPA5 fr;* *gltX fr*	3.04	3.36				
2207	NI				0.32		
2305	NI	0.21					
2402	NI						2.04
2405	*HNRNPF fr*	1.68	1.85				
2407	*HSPA5 fr;* *ALB fr.*	2.22					
2607	*TUBA1A fr;* Actin *fr*	2.08	2.38				
2608	*betaTub56D fr*	2.81					
2802	*actb*				0.32		
2806	NI				0.28		
3203	NI					0.28	
3303	*GSTP1;* *PRDX2;* *CRKL fr;* *EZR fr*	2.85	2.62				
3602	Tubulin *fr;* Actin	2.35					
3603	Actin *fr* *ACTN1 fr* *PDIA6 fr* Tubulin *fr* *NCL fr* *SRSF1*				0.45		
4405	*PKM fr* *PRDX4;* *ATP5F1B fr;*	2.07	1.85				
4608	*TCP1 fr;* *PKM fr*		2.29				
4801	*LUM*		0.17				
4807	NI	0.21					
5006	*S100A11*	5.50					
5105	*FABP1*	0.29	0.23				
5303	*HNRNPH1*/*HNRNPH2 fr.* *RAB11A/RAB11B*	3.04					
5307	*HNRNPH1*/*HNRNPH2 fr*; *COL1A2 fr*	3.13	2.67				
5603	*PKM fr*	2.03					
7106	*HBB*					0.17	

The figures given are detected significant fold changes of more than 1.5-fold (Wilcoxon signed rank test, *p* < 0.05). C, central part of tumor, P, peripheral part of tumor, N, non-involved part. NI, No protein identification. TNM_L_, Low stage TNM classification, TNM_H_, high stage TNM classification, see text and Table 1. *fr indicates that the theoretical mass is higher than the observed suggesting that the spot contains a fragment of the protein.

## Data Availability

The data presented in this study are available in this paper and supplementary file.

## Supplementary Materials

The following are available online at https://www.mdpi.com/article/10.3390/cimb43020074/s1, Table S1: Identification of spots separated by 2D-PAGE.
