# Supplementary material for: Proteomic Characterization of Colorectal Cancer Tissue from Patients Identifies Novel Putative Protein Biomarkers
Source: Curr Issues Mol Biol. 2021 Sep 2;43(2):1043–56. doi: 10.3390/cimb43020074 (PMC8929084; doi:10.3390/cimb43020074)
Supplement: Supplementary file 1 [file cimb-43-00074-s001.zip › cimb-1369988-supplementary.pdf]

Table S1. Identification of spots separated by 2D-PAGE

Spot	Protein identification	Gene	Peptides	Mr	Ion score	Protein name
0210	MYL6_HUMAN	*MYL6*	6	16919	513	Myosin light polypeptide 6
0212*	TBB2A_HUMAN** *fr*	*TUBB2A*	1 Ox (M)	App. 50000	69	Tubulin beta-2A chain
0611	A1AT_HUMAN *fr*	*SERPINA1*	2	46707	88	Alpha-1-antitrypsin
	ENPL_HUMAN *fr* or	*HSP90B1*	1	92411	82	Endoplasmin or
	HS90B_HUMAN *fr*	*HSP90AB1*		83212		Heat shock protein HSP 90-beta
0702	A1AT_HUMAN *fr*	*SERPINA1*	5	46707	536	Alpha-1-antitrypsin
1112	THIO_HUMAN	*TXN*	3	11730	183	Thioredoxin
1406	ALBU_HUMAN *fr*	*ALB*	6	69321	361	Albumin
1407	ACTB_HUMAN** *fr*	*ACTB*	2	41710	218	Actin, cytoplasmic 1
			3		237	
	ENOA_ HUMAN *fr*	*ENO1*	1	47139	89	Alpha-enolase
	ALBU_HUMAN *fr*	*ALB*	1	69321	49	Albumin
	FIBG_HUMAN *fr*	*FGG*	1	51479	56	Fibrinogen gamma chain
1604	ATPB_HUMAN *fr*	*ATP5F1B*	3	56525	238	ATP synthase subunit beta, mitochondrial
1605	ACTB_HUMAN** *fr*	*ACTB*	4	41710	305	Actin
1703	HSP7C_HUMAN *fr*	*HSPA8*	3	70854	169	Heat shock cognate 71 kDa protein
1806	HSP7C_HUMAN	*HSPA8*	6	70854	456	Heat shock cognate 71 kDa protein
			6		373	
	VIME_HUMAN *fr*	*VIM*	1	53695	70	Vimentin
	BIP_HUMAN *fr*	*HSPA5*	1	72288	113	Endoplasmic reticulum chaperone BiP
	SYE_ACIAD *fr*	*gltX*	1	57585	43	Glutamate-tRNA ligase (Acinetobacter sp.)
2207	NI					
2305	NI					
2402	NI					
2405	HNRPF_HUMAN *fr*	*HNRNPF*	2	45643	222	Heterogeneous nuclear ribonucleoprotein F
2407	BIP_HUMAN *fr*	*HSPA5*	5	72288	311	Endoplasmic reticulum chaperone BiP
	ALBU_HUMAN *fr*	*ALB*	2	69248	96	Albumin
2607	TBA1A_HUMAN** *fr*	*TUBA1A*	5	50000	394	Tubulin alpha-1A chain
	ACT_THELA *fr*	*N/A*	3	41609	257	Actin (Thermomyces lanuginosus)
2608	TBB1_DROME *fr*	*betaTub56D*	3	50115	242	Tubulin beta-1 chain (Drosophila melanogaster)
2802	ACTB_CTEID	*actb*	6	41726	447	Actin, cytoplasmic 1 (Ctenopharyngododon idella)
2806	NI					
3203	NI					
3303	GSTP1_HUMAN	*GSTP1*	3	23341	220	Glutathione S-transferase P
	PRDX2_HUMAN	*PRDX2*	3	21878	176	Peroxiredoxin-2
	CRKL_HUMAN *fr*	*CRKL*	1	33756	67	Crk-like protein
	EZRI_HUMAN *fr*	*EZR*	1	69370	47	Ezrin
3602	Tubulin** *fr*		9	50000	683	Tubulin
	Actin **		2	41976	177	Actin
3603	Actin ** *fr*		4	40000	299	Actin
			5		423	
	ACTN1_HUMAN	*ACTN1*	2	102993	95	Alpha-actinin-1
	PDIA6_HUMAN *fr*	*PDIA6*	1	48091	66	Protein disulfide-isomerase A6
			2		142	
	Tubulin** *fr*		1	50120	58	Tubulin
	NUCL_HUMAN *fr*	*NCL*	1	76568	55	Nucleolin
	SRSF1_HUMAN	*SRSF1*	2	27728	50	Serin/arginine-rich splicing factor
4405	PRDX4_HUMAN	*PRDX4*	5	30521	406	Peroxiredoxin-4
			5		448	
	ATPB_HUMAN *fr*	*ATP5F1B*	1	56525	57	ATP synthase subunit beta, mitochondrial
			3		204	
	KPYM_HUMAN *fr*	*PKM*	1	58009	45	Puruvate kinase PKM
4608	TCPA_HUMAN *fr*	*TCP1*	3	60306	118	T-complex protein 1 subunit alpha
	KPYM_HUMAN *fr*	*PKM*	2	57900	105	Puruvate kinase PKM
4801	LUM_HUMAN	*LUM*	1	38405	62	Lumican
4807	NI					
5006	S10AB_HUMAN	*S100A11*	5	11733	330	Protein S100-A11
5105	FABPL_HUMAN	*FABP1*	1	14199	35	Fatty acid binding protein, liver
5303	HNRH1/2_HUMAN *fr*	*HNRNPH1*	1	49198	117	Heterogeneous nuclear ribonucleoprotein H
		HNRNPH2		49232		Heterogeneous nuclear ribonucleoprotein H2
	RB11A/B_HUMAN	*RAB11A*	2	24378	116	Ras-related protein Rab-11A/
		RAB11B		24473		Ras-related protein Rab-11B
5307	HNRH1/2_HUMAN *fr*	*HNRNPH1*	1	49198	133	Heterogeneous nuclear ribonucleoprotein H
		HNRNPH2		49232		Heterogeneous nuclear ribonucleoprotein H2
	CO1A2_HUMAN *fr*	*COL1A2*	1	129235	83	Collagen alpha-2(I) chain
5603	KPYM_HUMAN *fr*	*PKM*	5	57900	343	Pyruvate kinase PKM
7106	HBB_HUMAN**	*HBB*	8	15988	670	Hemoglobin subunit beta

*Contaminants present besides the general as described in the text.

**Several variants found.

fr indicates that the molecular mass of the spot is lower than the theoretical.

Identifications, see: http://www.uniprot.org/uniprot/
Ion scores were calculated as -10Log(*p*). For explanation see:
http://www.matrixscience.com/help/interpretation_help.html
